# Hepatitis A outbreak since November 2016 affecting men who have sex with men (MSM) in Chile connected to the current outbreak in MSM in Europe, situation up to October 2017

**DOI:** 10.2807/1560-7917.ES.2018.23.9.18-00060

**Published:** 2018-03-01

**Authors:** Violeta Rivas, Aldo Barrera, Karla Pino, Ruth Núñez, C. Joaquin Caceres, Marcelo Lopez-Lastra, Alejandro Soza

**Affiliations:** 1 Departamento de Gastroenterología. Facultad de Medicina, Pontificia Universidad Católica de Chile, Santiago, Chile; 2 These authors contributed equally to this article; 3 Laboratorio de Virología Molecular, Instituto Milenio de Inmunología e Inmunoterapia, Centro de Investigaciones Médicas, Departamento de Enfermedades Infecciosas e Inmunología Pediátrica. Escuela de Medicina, Pontificia Universidad Católica de Chile, Santiago, Chile

**Keywords:** hepatitis A virus, outbreaks, men who have sex with men - MSM, Latin America, Chile

## Abstract

A hepatitis A outbreak has occurred in Chile since November 2016. Men are predominantly affected, with a large proportion of men who have sex with men (MSM). We describe 12 consecutive unrelated confirmed cases who presented at our healthcare institution in Santiago Metropolitan Area. Nine were men, all reporting having had sex with men. Ten viral sequences, genotyped as IA, clustered with the V16–25801 strain causing outbreaks mostly in MSM in Europe since mid-2016.

Hepatitis A virus (HAV) infection is usually transmitted by ingestion of contaminated food and/or water, but sexual transmission including among men-who-have-sex-with-men (MSM) has been described [1,2]. During 2016–2017, several HAV infection outbreaks, primarily involving MSM were reported in Europe [3-6]. The viruses responsible for these outbreaks were genotype IA and belonged to one of three main genetic clusters represented by the following respective strains: VRD_521_2016, RIVM-HAV16-090 and V16-25801 [7]. In the same period, HAV infection outbreaks affecting MSM were additionally reported from the United States (US) [8], as well as Israel where the VRD_521_2016 and RIVM-HAV16-090 strains were detected [5]. Since the end of 2016, a HAV infection outbreak has also taken place in Chile, which is disproportionally affecting men, and among them a large proportion of MSM. Herein, we describe the epidemiological characteristics of 12 patients with hepatitis A who sought care at the Red de Salud UC Christus, Santiago Metropolitan Area. A phylogenetic analysis of the HAV RNA sequences derived from 10 patients is also presented.

## Case definitions

The Chilean Ministry of Health surveillance criteria for HAV cases are [9]: (i) suspected case: compatible clinical features with elevated liver enzymes; (ii) confirmed case: suspected case plus a positive HAV specific test (anti-HAV IgM positive), or a suspected case with proven epidemiological link with a confirmed case in the preceding 15 to 50 days. 

For the purposes of our study, confirmed cases corresponded to both ambulatory and hospitalised patients (≥ 18 years-old) admitted at our university health network (Red de Salud UC Christus) with clinical symptoms compatible with acute hepatitis, compatible laboratory tests (alanine aminotransferase (ALT) greater than 10 times the upper limit of normal – 30 U/mL – and/or elevated bilirubin level > 1 mg/dL), and a positive anti-HAV IgM from 1 June to 31 October 2017.

## Description of the outbreak in Chile

The outbreak in the country started in November 2016 and between 1 January 2017 and 7 October 2017, a total of 2,227 confirmed cases according to the Ministry of Health definition were notified (cumulated incidence of 12.1 cases per 100,000 habitants) [10]. Cases peaked in mid-2017 (epidemiological week 20), corresponding to a 168% increase in HAV cases in Chile compared with 2016. Specifically, in the Santiago Metropolitan Area the number of cases rose from 91 to 1,257 (incidence increased from 1.2 to 16.8 infections per 100,000 habitants) from 2016 to 2017, respectively. The median number of cases from 2009 to 2015 in this area was 131.

Interestingly, the current Chilean HAV infection outbreak affects men in a 5:1 ratio (17:1 ratio in the 25–29 age range) and 72% are in the 20 to 39 years of age range. According to the epidemiological investigation of the Ministry of Health, most have university studies (57%), and live in communes with medium or high income. Among affected men, 61% have had sex with men and 32% have a previous history of sexually transmitted infection (mostly syphilis and human immunodeficiency virus (HIV)) [11]. In terms of disease severity, 20% of patients required hospitalisation and to our knowledge at least one patient required a liver transplant (data not shown).

## Studied patients

Here we provide the epidemiological and molecular description of 12 consecutive confirmed cases (according to the study case definition) who sought care at our institution located in the Santiago Metropolitan Area. A clinical and epidemiological questionnaire was applied by the investigator. A blood sample was obtained at the time of interview. Patients provided a written informed consent to participate in the study. This study was approved by the Scientific Ethics Committee of the Pontificia Universidad Católica de Chile (study N° 14–019).

Epidemiological features of the patients are summarised in the Table. All cases were unrelated. Briefly, nine of 12 were men, all of whom declared to have had sex with men during the last year. The three women had close contact with MSM, whereby they were either a relative of, or living under the same roof as an MSM. Five patients were HIV positive, of whom two also had syphilis and one chronic hepatitis B. Clinical presentation was classical (fatigue, nausea, choluria, jaundice), but four patients required hospitalisation. One patient had received HAV vaccine (Twinrix: 3 doses administered on a 0-, 1- and 6-months schedule, according to the manufacturer’s guidelines) in 2010 and a second patient had a positive anti-HAV IgG antibody in 2014 (so HAV vaccine was not prescribed at the time). Both were HIV positive with treated and controlled disease (undetectable HIV RNA and CD4 count > 500 cells/mm^3^).

**Table:** Demographic, risk factors, underlying disease, and liver function tests from cases of acute hepatitis A in the Santiago Metropolitan Area, Chile (n = 12)

Characteristic	Total (n:12)
**Demographic characteristics**	
Age, median (range)	32 (21–56)
Men, number	9
**Epidemiological characteristics and risk factors**	
Foreigners, number**^a^**	1
MSM/men, ratio	9/9
High-risk sexual behaviour^b^, number	9
Contact with infected individuals, number**^c^**	3
Travelled in the 2 months before symptom onset, number	0
Cases who used drugs^d^, number	5
Cases with severe hepatitis**^e^**, number	2
Cases which were hospitalised, number	4
**Sexually transmitted co-infections**	
HIV, number	5
HBV, number	1
HCV, number	0
Syphilis, number	2
**Liver function tests **	
ALT, median (range)	3,609 (1,615–5,904)
Total bilirubin, median (range)	8.6 (4–21)
INR, median (range)	1.4 (1.2–2.0)

ALT: alanine aminotransferase; HBV: hepatitis B virus; HCV: hepatitis C virus; HIV: human immunodeficiency virus; INR: international normalised ratio; MSM: men who have sex with men; SD: standard deviation.

^a^ One case was from Venezuela.

^b^ Including unprotected sex and anal sex.

^c^ The three patients who reported having close contact with somebody with known acute hepatitis A were women.

^d^ Two patients consumed cocaine, four cannabis, and one poppers (alkyl nitrites). None of them reported intravenous drug use.

^e^ Severe hepatitis was defined as an INR ≥1.5. None of them presented with hepatic encephalopathy.

## Phylogenetic analysis

Total viral RNA was extracted from 10 patients’ samples respectively and amplified by RT-PCR generating a 406 nt amplicon comprising the viral protein 1/protease 2A (VP1/P2A) region of the HAV genome. Each amplicon was sequenced according to the HAVNET shared protocol [12] and the obtained sequences deposited in HAVNET under the following accession numbers: Chile-P1 to Chile-P9 and Chile-P11. Results of a phylogenetic analysis (Figure) showed that the strains affecting the 10 patients were all of genotype 1A, with their sequences closely grouping with that of the V16–25801 strain. The sequence cluster represented by V16–25801 was originally reported in Germany in January 2017, but cases affected by strains of this cluster have also been reported from the United Kingdom and Spain [7,13]. Interestingly, a retrospective analysis showed that two patients in Italy with strains belonging to the V16–25801 cluster in 2014 and 2016 were from Ecuador, suggesting that the V16–25801 strain could have been circulating in South America prior to the European outbreak [7].

**Figure:**
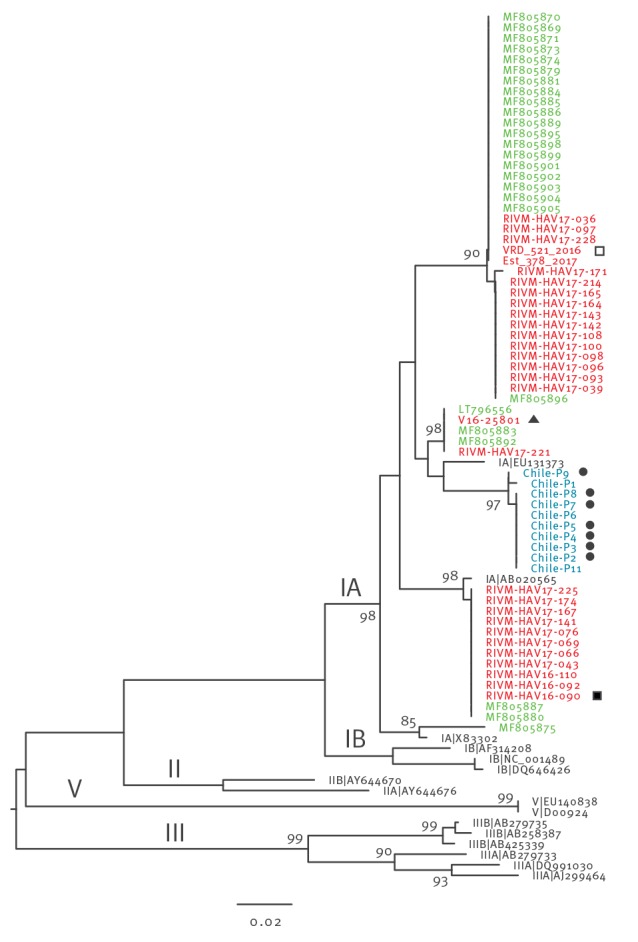
Phylogenetic analysis of the VP1/P2A genomic region (346 nt) of viral strains from hepatitis A cases in Chile, 2017

Consensus sequences from 10 patients participating in the current study (blue) were aligned by Clustal W, to sequences obtained between 2016 and 2017 from European (red) and Spanish (green) cases associated to men who have sex with men (MSM), plus prototype sequences of each genotype (indicated by roman numerals (black)). The non-Spanish European sequences (red) were obtained from the RIVM HAVNET database (https://rivm.nl/en/Topics/H/HAVNET/Database) and are described by their HAVNET accession numbers. The prototype and Spanish sequences were retrieved from GenBank. For the prototype sequences, the genotypes are depicted followed by the GenBank accession numbers in black, while for the Spanish sequences, only the GenBank accession numbers figure in green. The green colour used to distinguish Spanish sequences from other European sequences in red, was to allow better visualisation of the relationships between the Spanish and Chilean sequences, given the close ties between Chile and Spain, which promote contact of people and travel activities between the two countries. The phylogenetic tree was constructed by maximum likelihood method based on the Tamura–Nei model, and bootstrap values of 1,000 replicates (> 80) are indicated at the nodes. MSM-associated Chilean strains (closed circles), and prototype strains of the three European outbreak clusters (the RIVM-HAV16-090 which was detected at the******2016 Europride: closed square; the VRD_521_2016 which was detected in the******United Kingdom and later in Spain: open square; and the V16-25801 which was detected in Germany: triangle) are indicated. The scale bar shows genetic distance (substitution per nt position). Evolutionary analysis was conducted in MEGA7.

## Control measures

The Chilean Ministry of Health has instituted several measures for controlling the current outbreak, including reinforcing the diagnostic capabilities of the public health system, increasing immunisation for close contacts (under the age of 40 years) of hepatitis A cases, education to targeted groups (MSM), working with social organisations and offering free vaccination for all HIV infected patients younger than 40 years-old [10].

## Discussion

Chile is transitioning from a high to a low endemicity in HAV infection according to the World Health Organization definitions [14]. This is linked to periodical outbreaks and a paradoxical raise in acute HAV infection in young people and more severe disease, with hepatitis A being the leading cause of acute liver failure in both Chile and a number of other countries in Latin America [15]. In this study, we report on a large Chilean HAV infection outbreak, showing epidemiological features similar to the ongoing outbreak in Europe, that is, affecting mostly young men and among these a high proportion of MSM (which is new in this region). Our data on a subset of patients infected with HAV show that sequences of the viruses from these patients correspond to genotype 1A and are related to one of the three main strains described in Europe in the context of HAV infection outbreaks in MSM (V16–25801 cluster). This suggests that the current outbreak affecting Europe is also occurring in Latin America.

It is not clear from our data what the specific epidemiological link between the Chilean and European outbreak is, since none of our patients had a history of travel to Europe. In a study conducted between January and June 2017, which investigated the HAV infection outbreak in Barcelona, Spain, one of the patients reported a history of travel to Chile [6]. Unfortunately, the genetic material from the sample of this particular patient did not amplify by PCR (personal communication, Sergio Rodríguez-Tajes, December, 2017), making a direct viral sequences comparison impossible. Moreover, the possibility that the V16–25801 cluster may have originated from South America and subsequently propagated to Europe cannot be ruled out.

A major weakness of our study is the limited number of cases investigated, which restricts the generalisation of the results. Nevertheless, the clinical characteristics of the described consecutive patients are consistent with the characteristics of the outbreak (young, MSM) and the viral sequences derived from 10 of these patients belong to a single cluster.

Following the advice of the Chilean Society of Infectology [16], which was prompted by the current outbreak, a single dose of inactivated HAV vaccine for all infants at age 18 months has been incorporated to the Chilean Universal Expanded Immunization Programme. The programme considers a local cost-effectiveness analysis [17] and the good results of single dose immunisation in children in Latin America [18,19]. We hope that in the long term this public health intervention will protect the future generations from outbreaks like the one we are now experiencing.

In our view, it is crucial for countries in the epidemiological transition from high to low endemicity (such as many countries in Latin America) to quickly recognise and differentiate endemic outbreaks in which the traditional food-borne route is involved, from outbreaks related to sexual transmission in MSM. Different control measures and approaches are required depending on the type of outbreak. In addition to vaccination of close contacts and passive immunisation, HAV vaccination for MSM (not only for HIV positive individuals) seems to be a critical measure. Sequencing and analysis of the strains responsible for the outbreaks may be helpful for understanding and planning a public health response.

In conclusion, the large hepatitis A infection outbreak in Chile is closely linked to the current outbreak in 19 European countries. As many cases in MSM with closely related strains have been described in the east coast of the US [8], Israel [5] and now in Chile, it seems that the current HAV infection outbreak affects a larger geographical area than Europe.
